# Predicting the subcellular localization of viral proteins within a mammalian host cell

**DOI:** 10.1186/1743-422X-3-24

**Published:** 2006-04-04

**Authors:** MS Scott, R Oomen, DY Thomas, MT Hallett

**Affiliations:** 1 McGill Center for Bioinformatics, McGill University, 3775 University Street, Montreal, Quebec, Canada; 2 Integrated Genomics, Sanofi Pasteur, 1755 Steeles Avenue West, Toronto, Ontario, Canada; 3 Biochemistry Department, McGill University, McIntyre Medical Sciences Building, 3655 Promenade Sir William Osler, Montreal, Quebec, Canada

## Abstract

**Background:**

The bioinformatic prediction of protein subcellular localization has been extensively studied for prokaryotic and eukaryotic organisms. However, this is not the case for viruses whose proteins are often involved in extensive interactions at various subcellular localizations with host proteins.

**Results:**

Here, we investigate the extent of utilization of human cellular localization mechanisms by viral proteins and we demonstrate that appropriate eukaryotic subcellular localization predictors can be used to predict viral protein localization within the host cell.

**Conclusion:**

Such predictions provide a method to rapidly annotate viral proteomes with subcellular localization information. They are likely to have widespread applications both in the study of the functions of viral proteins in the host cell and in the design of antiviral drugs.

## Background

Viruses use the host synthetic machinery to replicate. They have evolved mechanisms to exploit the host nucleic acid replication and protein translation apparatus and have also developed strategies to evade humoral immune surveillance. Viral proteins require targeting to the appropriate subcellular compartments of the host cell to fulfill their roles. Viral proteins have been shown experimentally to be localized in many different cellular compartments including the nucleus (for example the protein kinase encoded by Epstein-Barr Virus [1]), the nucleolus (such as the rev and tat proteins from human immunodeficiency virus type 1 [2]), the cytosol (for example the superoxide dismutase-like protein from vaccinia virus [3]), the ER/Golgi apparatus (for example, the US2 and US11 cytomegalovirus proteins [4,5]), the plasma membrane and cell surface (cytomegalovirus gp34 glycoprotein [6]), and the mitochondria (M11L protein from the myxoma virus and several others, reviewed in [7,8]). Targeting to the extracellular space is also observed (for example, cowpox growth factor [9] and the myxoma M-T7 protein [10]).

Protein subcellular localization prediction has been widely studied (reviewed in [11,12]). Available predictors differ in many aspects including the computational method used, the type and diversity of protein characteristics considered for the prediction, the localization coverage, the target organism(s) and the reliability. Predictors can be grouped into four general classes based upon the protein characteristics that are considered: amino acid composition and order based predictors [13-15], sorting signal predictors [16,17], homology based predictors [18,19] and hybrid methods that integrate several sources of information to predict localization [20-23].

Although numerous protein localization predictions exist for whole prokaryotic and eukaryotic proteomes, no such predictions are available for many viral proteins, which are often involved in extensive interactions with host proteins in various subcellular localizations in the host cell. This is surprising as such predictions would be of great use in the study of infectious diseases in order to increase our understanding of the role of these proteins in host cells and could also be useful for the design of improved therapeutic interventions.

Here, we investigate the intracellular localization predictions of viral proteins in human cells. We focus on two viruses, vaccinia virus and human cytomegalovirus, because they infect human cells and have relatively large but well characterized genomes. We show that these viral proteomes harbour many known eukaryotic targeting signals and domains which probably allow them to exploit cellular localization mechanisms. We also use the PSLT human localization predictor [22] to demonstrate that an appropriately chosen predictor can accurately predict the intracellular localization of viral proteins in human cells. Our viral subcellular localization predictions are available as additional files.

## Results

### Eukaryotic targeting signals and functional domains in specific viral proteomes

In order to investigate the extent of eukaryotic targeting signal usage by the viral proteins considered, we scanned the human, vaccinia virus and cytomegalovirus proteomes using various bioinformatics predictors that identify these signals. To avoid redundancy in the datasets, we considered all proteins available in UniProt [24] from one representative strain of each virus (we chose the AD169 strain for the cytomegalovirus and the Copenhagen strain for the vaccinia virus). As shown in Table 1, despite differences in genome size of several orders of magnitude, several targeting signals are found to a similar extent in both viral and human proteomes. In particular, large numbers of these viral proteins contain N-terminal signal peptides and anchors, consistent with the knowledge that many glycoproteins encoded in these large viruses require entry into the secretory pathway and have evolved to modulate ER quality control mechanisms to ensure that large quantities of viral proteins can be correctly produced and assembled into infectious particles [25]. Similarly, a high proportion of viral proteins are predicted to contain at least one transmembrane domain. This reflects the high degree of interaction of these enveloped viruses with cellular membranes for functions that include assembly of viral particles and budding of the virus [26], and thus the need for insertion of a large proportion of their proteins in membranes, to participate in and modulate these processes. The vaccinia virus and cytomegalovirus proteomes also contain proteins that are predicted to harbor mitochondrial targeting peptides. Both cytomegalovirus and vaccinia virus are known to encode at least one protein that is localized to mitochondria, where they play a role in the inhibition of apoptosis [7]. GPI anchors, which allow the attachment of proteins to the extracellular leaflet of the plasma membrane, are also predicted to be used by these viral proteins, to a similar extent as by human proteins. This might constitute a significant viral localization mechanism. In contrast to the relatively large proportion of viral proteins harbouring a C-terminal GPI-attachment region, very few of these viral proteins are predicted to be prenylated, which might reflect a greater need for extracellular rather than intracellular anchoring of these viral proteins in the plasma membrane.

**Table 1:** Usage of targeting signals in human and viral proteins

**Organism**	**Human**	**Cytomegalovirus **(strain AD169)	**Vaccinia **(Copenhagen strain)	
**Protein count**	28908	192	255	

**Targeting signals**	**Percentage of proteins containing signal**			**Predictor**
Signal peptide	22.0%	25.0%	12.9%	SignalP [16]
Signal anchor	5.9%	8.8%	5.9%	SignalP
Mitochondrial targeting peptide	8.5%	12.0%	7.1%	Predotar [40]
0 TMD^a^	78.0%	66.1%	76.5%	TMHMM [41]
1 TMD^a^	10.0%	19.8%	16.1%	TMHMM
2 TMDs^a^	2.4%	5.7%	5.9%	TMHMM
>2 TMDs^a^	9.7%	8.3%	0.8%	TMHMM
GPI anchor	2.5–3.0%^b^	3.1%	2.4%	GPI-SOM [42]
Prenyl group (PS00294)	0.8%	0.5%	0%	Prosite [43]
NLS	13.3%^c^	8.3%	2.0%	PredictNLS [44]
KDEL-like (PS00014)	0.2%	0%	0%	Prosite
Peroxisomal targeting (PS00342)	2.2%	1.6%	2.8%	Prosite

**Most prevalent eukaryotic functional domains**	**Percentage of proteins containing domain**			**Predictor**
Immunoglobulin-like (IPR007110)	2.7%	5.7%	2.0%	InterPro [28]
Galactose oxidase (IPR011043)	0.3%	0%	1.6%	InterPro
Proteinase inhibititor I4, serpin (IPR000215)	0.2%	0%	1.6%	InterPro
Rhodopsin-like GPCR superfamily (IPR000276)	2.8%	2.1%	0%	InterPro

^a ^TMD: transmembrane domain

^b^estimation for proportion of human proteins containing a GPI anchor from [42]

^c^estimation for proportion of human proteins containing an NLS (nuclear localization signal) from [44].

Nuclear localization signals (NLSs) can also be detected in the viral proteomes. A larger proportion of cytomegalovirus proteins are predicted to contain NLSs than those encoded by the vaccinia virus genome. This is consistent with the fact that the cytomegalovirus genome replication as well as its viral core and capsid assembly occur in the nucleus whereas the vaccinia virus coordinates these processes in the cytoplasm.

We also detected the presence of short targeting signals in these proteomes. The N-terminal KDEL-like endoplasmic reticulum (ER) retrieval motif that is present in approximately 20% of human ER lumenal proteins [27] does not seem to be used by these viral proteins but the highly non-specific peroxisomal-targeting signal is present to the same extent in these viral and human proteins.

The most prevalent functional eukaryotic domains present in these viral proteins are also shown in Table 1, as predicted by InterPro [28]. The immunoglobulin-like domain is the most widely used eukaryotic domain in both cytomegalovirus and vaccinia virus, which are well known to extensively modulate the immune response of the host (reviewed in [29,30]). The galactose oxidase and proteinase inhibitor I4 domains are over-represented in vaccinia virus but absent in cytomegalovirus suggesting that these domains are not used as part of a viral strategy common to these two viruses but are rather specific to vaccinia virus. Similarly, the rhodopsin-like GPCR superfamily is prevalent in cytomegalovirus proteins but absent from vaccinia virus. Cytomegalovirus is known to encode at least four G-protein coupled receptors, which could allow it to modulate and antagonize host signalling pathways [31].

Interestingly, protein-protein interaction domains such as SH2, SH3, WW and t-snare domains are conspicuously absent from these viral proteomes (data not shown), indicating that mimicry and modulation of this type of cellular communication mechanism might not be part of the survival strategy of these viruses.

The very high proportion of viral proteins containing one or several eukaryotic targeting motifs and functional domains shows the extensive usage of cellular localization mechanisms and machinery by these viruses. This provides a good indication that eukaryotic protein subcellular localization predictors might perform well on these viruses.

### Subcellular localization prediction of viral proteins in host cells

We used the PSLT human subcellular localization predictor [22] to predict the localization of cytomegalovirus and vaccinia virus proteins and to investigate whether principles of eukaryotic protein localization prediction can be applied to viral proteins. PSLT is a Bayesian network type tool, trained on human sequences, that predicts the subcellular localization of proteins based on the co-occurrence of protein domains, motifs and targeting signals. Table 2 shows the predictions of vaccinia virus proteins whose cellular localization has already been studied experimentally and is available in the literature (the full prediction dataset is available as supplementary material, please see Additional file 1). As shown in Table 2, the localization of most vaccinia virus proteins is well-predicted. The accuracy of PSLT on this dataset can be estimated to be 78% (proteins localized to more than one compartment are considered to be accurately predicted if at least one predicted compartment agrees with the previous literature annotation). A large proportion (36%) of these proteins are predicted to be secreted or expressed on the cell surface as integral membrane proteins or membrane anchored proteins. For the most part, this prediction is confirmed in the literature, but it should be considered a conservative estimate, since experimental studies cannot always sample the kinetics of viral protein synthesis and trafficking in all systems under all conditions. This estimate of extracellular and cell surface viral proteins is higher than our estimate of 22% for human cellular proteins [22], and likely reflects important viral functions that require using the host secretory pathway. Indeed, several of these proteins are known to be involved in modulating the host immune response including secreted proteins that bind chemokines, interferons and interleukin family members [30,32]. Other such proteins are incorporated in the viral envelope. Few or no vaccinia proteins are predicted to localize to the peroxisome, lysosome, ER or Golgi apparatus.

**Table 2:** Subcellular localization prediction of vaccinia virus proteins

**Gene**	**SwissProt Accession**	**PSLT predictions^a^**	**Literature annotations**	**Localization References**	**Closest human homologue^b^**	**BLAST e-value**
*A34R*	P21057	PM	Golgi	[45]	C-type lectin (NP_072092)	0.02
*A38L*	P21061	PM	PM	[46]	CD47 antigen (NP_001768)	3E-22
*A39R*	P21062	Secreted and PM	Secreted	[47]	semaphorin 7A (NP_003603)	1E-30
*A40R*	P21063	PM	PM	[48]	lectin-like receptor (NP_031359)	2E-9
*B13R*	P20841	Secreted	Cytoplasmic	[49]	plasminogen activator inhibitor-2 (NP_002566)	5E-7
*B15R*	P21116	Secreted and PM	PM or secreted	[50]	interleukin 1 receptor (NP_775465)	4E-32
*B18R*	P21076	Cytosolic and nuclear	Secreted and PM	[51]	Ankyrin 3 isoform 1 (NP_066267)	1E-9
*B5R*	P21115	Secreted and PM	PM and Golgi	[45,52,53]	coagulation factor XIII B (NP_001985)	6E-17
*C12L*	P20531	Secreted	Secreted	[54]	serine proteinase inhibitor (NP_002965)	4E-47
*C2L*	P21037	Nuclear and cytosolic	Cytoplasmic	[55]	kelch-like 10 (NP_689680)	1e-024
*D8L*	P20508	PM	PM	[56]	carbonic anhydrase XIII (NP_940986)	8E-35
*A45R*	P21132	Cytosolic	Cytoplasmic	[3]	superoxide dismutase 1 (NP_000445)	3E-6
*D4R*	P20536	Nuclear and mitochondrial	Cytoplasmic	[56]	-	> 0.1
*D6R*	P20634	Nuclear	Perinuclear	[58]	hypothetical protein (NP_060139)	0.027
*E9L*	P20509	Nuclear	Cytoplasmic	[57]	polymerase alpha (NP_058633)	9E-19
*E3L*	P21081	Nuclear	Nuclear	[59]	adenosine deaminase (NP_001102)	4E-6
*F17R*	P68454	Cytosolic and nuclear	Cytoplasmic	[60]	-	> 0.1
*F4L*	P20493	Cytosolic	Cytoplasmic	[57]	ribonucleotide reductase M2 (NP_001025)	1E-143
*A56R*	P20978	PM	PM	[61]	dentin matrix acidic phosphoprotein (NP_004398)	0.015
*K1L*	P20632	Cytosolic and nuclear	Cytoplasmic	[62]	ankyrin 2 (NP_001139)	1E-9
*K2L*	P20532	Secreted	Extracellularly associated with infected cell	[63]	plasminogen activator inhibitor-1 (NP_000593)	3E-35
*M1L*	P20640	Cytosolic and nuclear	Cytoplasmic	[64]	ankyrin 3 (NP_066267)	4E-13
*C11R*	P20494	PM and secreted	Secreted	[65]	epiregulin (NP_001423)	2E-10

^a ^In the case of multi-compartmental proteins (proteins that are predicted with high probability to be present in more than one compartment), the two most likely compartments were retained by PSLT. PM: plasma membrane.

^b ^The closest human homologue was determined by using BLAST [38] against the NCBI human RefSeq dataset. We do not report a homologue when the BLAST e-value exceeds 0.1.

Table 3 shows the PSLT predictions for cytomegalovirus proteins whose cellular localization has already been studied experimentally and is available in the literature (the full prediction dataset is available as supplementary material, please see Additional file 2). The prediction accuracy of PSLT on this dataset is estimated to be 60% according to the literature. Almost all proteins classified as wrongly predicted according to the literature are annotated as localized in the ER or Golgi apparatus but predicted by PSLT as being on the cell surface. Several of these proteins display characteristics of cell surface or secreted proteins such as the capability to bind MHC class I and class II antigens. However, instead of being secreted, these cytomegalovirus proteins localize to the ER where they bind the MHC antigens, effectively targeting them for degradation and leading to the protection of cytomegalovirus-infected cells from CD8+ and CD4+ T lymphocytes [33]. Many other cytomegalovirus proteins are well-predicted including cell surface receptors, several of which mimic host receptors [34] as well as secreted proteins such as viral chemokine and IL-10 homologues [35,36].

**Table 3:** Subcellular localization prediction of cytomegalovirus proteins

**Gene**	**SwissProt Accession**	**PSLT predictions^a^**	**Literature annotations**	**Localization References**	**Closest human homologue^b^**	**BLAST e-value**
*gp34*	P16809	PM	PM	[6]	-	> 0.1
*UL111A*	P17150	Secreted	Secreted	[36]	interleukin 10 (NP_000563)	2E-9
*UL114*	P16769	Nuclear	Nuclear	[66]	uracil-DNA glycosylase (NP_550433)	5E-39
*UL118/119*	P16739	PM	PM	[6]	-	> 0.1
*UL18*	P08560	PM/secreted	PM	[67]	MHC class Ib antigen (NP_005507)	4E-16
*UL33*	P16849	PM	PM, endosomes, secretory pathway, perinuclear	[34,68]	chemokine receptor 1 (NP_001286)	3E-21
*UL48*	P16785	Cytosolic and ER	ER, cytosolic, vacuolar	[69,70]	spen homolog, transcriptional regulator (NP_055816)	0.082
*UL54*	P08546	Nuclear	Nuclear	[71]	polymerase delta 1 (NP_002682)	3E-50
*UL78*	P16751	PM	PM	[34]	somatostatin receptor 3 (NP_001042)	0.006
*UL97*	P16788	PM, cytosolic	Golgi, nuclear and cytosolic	[72]	-	> 0.1
*US10*	P09728	PM	ER	[33]	-	> 0.1
*US11*	P09727	PM	ER	[4]	-	> 0.1
*US2*	P09713	PM	ER and cytosolic	[5]	-	> 0.1
*US27*	P09703	PM	PM, endosomes, secretory pathway, perinuclear	[34,68]	chemokine receptor 1 (NP_001328)	7E-31
*US28*	P69332	PM	PM, endosomes	[34,68]	chemokine receptor 1 (NP_001328)	2E-55
*US3*	P09712	PM	ER	[73,74]	-	> 0.1
*US6*	P14334	PM	ER	[75]	-	> 0.1
*US7*	P09731	PM	ER	[33]	-	> 0.1
*US8*	P09730	PM	Golgi	[33]	-	> 0.1
*US9*	P09729	Secreted	ER	[33]	-	> 0.1

^a ^In the case of multi-compartmental proteins (proteins that are predicted with high probability to be present in more than one compartment), the two most likely compartments were retained by PSLT. PM: plasma membrane; ER: endoplasmic reticulum.

^b ^The closest human homologue was determined by using BLAST [38] against the NCBI human RefSeq dataset. We do not report a homologue when the BLAST e-value exceeds 0.1.

We investigated whether the prediction accuracy of PSLT was correlated with the degree of similarity between the viral proteins and their closest human homologue. The two rightmost columns of Tables 2 and 3 show the closest human homologue from the NCBI RefSeq [37] database for each viral protein, as determined by BLAST [38]. In general, viral proteins that have close human homologues (BLAST e-value <= 1e-10) are accurately predicted by PSLT. The prediction accuracy for these proteins is 100% for the cytomegalovirus and 91% for the vaccinia virus. Some viral proteins that do not have close human homologues (BLAST e-value > 1e-10) are well-predicted but the overall prediction accuracy of PSLT for these proteins is lower (43% for cytomegalovirus proteins and 67% for vaccinia virus proteins). This is consistent with previous analyses which allowed us to show that the prediction accuracy of PSLT is greater when predicting proteins from organisms that are evolutionarily close to those used to train the predictor [22].

## Discussion

The proteomes of vaccinia virus and cytomegalovirus display numerous examples of eukaryotic targeting signals and functional domains, consistent with their evolutionary origin and their extensive usage of many elements of the host cellular machinery. We show here that, as a consequence, the subcellular localization of these viral proteins can be accurately predicted by human protein localization predictors. We used the PSLT predictor which considers the combinatorial presence of domains and targeting signals in human proteins to predict localization. This predictor might be better-suited for this task than other types of localization predictors. Indeed, PSLT specifically focuses on the localization of human proteins and has been shown to accurately predict the localization of mammalian proteins in general and thus is likely an appropriate choice for the prediction of the localization of viral proteins within human cells. Another advantage of PSLT is that it considers domains and motifs rather than amino acid composition. Many of these domains and motifs are likely involved in interactions with host proteins and should thus more closely resemble human sequences than other regions of the proteins. In fact, several of these domains are believed to have been stolen by these large viruses from host cells [39]. Viral-specific proteins might have evolved to resemble host protein motifs, in order to use mechanisms available in host cells. Not surprisingly, viral proteins that have a high degree of similarity to human proteins are generally better predicted than those that do not have a close human homologue. More extensive research into viral subcellular localization prediction will likely lead to higher prediction accuracy and coverage as the specific non-eukaryotic characteristics of viral proteins can also be exploited to determine their cellular localization. This will likely be particularly important to predict the localization of viral proteins that have little similarity to mammalian proteins.

## Conclusion

This study demonstrates that eukaryotic protein subcellular localization predictors can be used to rapidly annotate specific viral proteomes with a first and reasonably accurate estimate of intracellular localization. The subcellular localization prediction of viral proteins within human cells should be of great utility to the biological community to increase our understanding of the function of these proteins, of their role in the cell and of the consequences of host-pathogen interactions. They might also serve to devise more efficient methods of treatment by rapid identification of targets.

## Methods

28908 human protein sequences were retrieved from the Hera database [27]. These proteins represent all NCBI RefSeq [37] entries currently present in Hera. Cytomegalovirus and vaccinia virus protein sequences were downloaded from UniProt [24]. All sequences were scanned with the different predictors referred to in Table 1, using the default parameters.

The localization of the viral proteins was predicted using PSLT as previously described [22]. PSLT is a Bayesian network type tool that predicts the subcellular localization of proteins based on the co-occurrence of protein domains, motifs and targeting signals. PSLT was trained on human proteins as described in [22]. In the case of multi-compartmental proteins (proteins that are predicted with high probability to be present in more than one compartment), the two most likely compartments were retained. The closest homologue of all viral proteins in Tables 2 and 3 was determined by using BLASTP version 2.2.12 [38] against the NCBI human RefSeq dataset (release 15) [37]. The default parameters of BLASTP were used.

## Supplementary Material

Additional File 1 which contains protein localization predictions for several different strains of the vaccinia virus. Click here for file

Additional File 2 which contains protein localization predictions for several different strains of the human cytomegalovirus Click here for file
